# Cartilage-binding antibodies initiate joint inflammation and promote chronic erosive arthritis

**DOI:** 10.1186/s13075-020-02169-0

**Published:** 2020-05-24

**Authors:** Yanpeng Li, Dongmei Tong, Peibin Liang, Erik Lönnblom, Johan Viljanen, Bingze Xu, Kutty Selva Nandakumar, Rikard Holmdahl

**Affiliations:** 1 SMU-KI United Medical Inflammation Center, School of Pharmaceutical Sciences, Southern Medical University, Guangzhou, 510515 China; 2 Medical Inflammation Research, Department of Medical Biochemistry and Biophysics, Karolinska Institute, SE-17177 Stockholm, Sweden; 3 Department of Chemistry Biomedical Center, Uppsala University, Box 576, SE-75123 Uppsala, Sweden

**Keywords:** Rheumatoid arthritis, Cartilage, Animal models, Antibodies, Collagen

## Abstract

**Background:**

Antibodies binding to cartilage proteins are present in the blood and synovial fluid of early rheumatoid arthritis patients. In order to develop animal models mimicking the human disease, we have characterized the arthritogenic capacity of monoclonal antibodies directed towards different joint proteins in the cartilage.

**Methods:**

Purified antibodies specific to unmodified or citrullinated collagen type II (CII), collagen type XI (CXI), and cartilage oligomeric matrix protein (COMP) were produced as culture supernatant, affinity purified, pooled as antibody cocktails (Cab3 and Cab4), and injected intravenously into mice to induce arthritis. An adjuvant (lipopolysaccharide or mannan) was subsequently injected intraperitoneally on either day 5 or day 60 to enhance arthritis. Antibody binding and complement activation on the cartilage surface were analyzed by immunohistochemical methods. Bone erosions and joint deformations were analyzed by histological assessments, enzyme-linked immunosorbent assays, and micro-CT. Luminex was used to detect CII-triple helical epitope-specific antibody responses.

**Results:**

The new cartilage antibody cocktails induced an earlier and more severe disease than anti-CII antibody cocktail. Many of the mouse strains used developed severe arthritis with 3 antibodies, binding to collagen II, collagen XI, and cartilage oligomeric matrix protein (the Cab3 cocktail). Two new models of arthritis including Cab3-induced LPS-enhanced arthritis (lpsCAIA) and Cab3-induced mannan-enhanced arthritis (mCAIA) were established, causing severe bone erosions and bone loss, as well as epitope spreading of the B cell response. Cab4, with addition of an antibody to citrullinated collagen II, induced arthritis more efficiently in moderately susceptible C57BL/6 J mice.

**Conclusions:**

The new mouse model for RA induced with cartilage antibodies allows studies of chronic development of arthritis and epitope spreading of the autoimmune response and bone erosion.

## Background

Autoimmune diseases, such as rheumatoid arthritis (RA) and systemic lupus erythematosus (SLE), develop in three distinct stages: priming, onset, and chronicity [1]. These autoimmune diseases are mainly associated with two types of genes. It is well known that the major histocompatibility complex class II (MHC II), in particular certain DR alleles, are the most important genes in the so called seropositive or classical RA, characterized by development of autoantibodies in serum [1, 2]. The identity of the MHC class II protein or its function in the disease is not conclusively known, but the association gives a strong indication that autoreactive T cells are involved early in the pathogenesis. The MHC class II region is associated with an IgG antibody response to post-translationally modified proteins, which requires the activation of autoreactive T cells [3]. A more recent discovery shows that the polymorphism of the neutrophil cytosolic factor 1 (*Ncf1*) gene is a major genetic factor in animal models of autoimmune diseases [4, 5]. The *Ncf1* locus has so far not been included in genome-wide association studies due to extensive and variable duplications of the *Ncf1* gene in humans. However, both a specific single-nucleotide polymorphism leading to lower reactive oxygen species (ROS) production and a copy number variation polymorphism are associated with SLE and RA [6–8].

In classical RA, priming is characterized by the activation of B cells to produce disease-specific IgG autoantibodies that may appear in the blood several years before clinical onset of the disease [9]. These include antibodies directed towards modified IgG (rheumatoid factors, RF), anti-citrullinated protein antibodies (ACPA), and antibodies to other forms of modifications such as lysine and arginine side-chains, including antibodies to carbamylated proteins [10–12], which all predict disease development. However, how this priming stage becomes an inflammatory attack on the joints, leading to clinical onset, remains unknown. B cells are likely to play a pathogenic role in early established RA, as shown by the finding that depletion of CD20^+^ B cells with rituximab has a therapeutic effect [13]. In early RA, both seronegative and seropositive, a diverse set of antibodies to various cartilage proteins including type II collagen (CII) can be detected [14–16]. A high affinity antibody response to a cartilage protein like triple helical CII can be detected only in a few percentage of patients, but since it is possible that reactivities to different cartilage proteins, including their modifications like citrullination or carbamylation, and the time period in which they appear are not yet known, it is likely to be seen more commonly.

Monoclonal antibodies to CII induce arthritis after injection into mice [17, 18], and these antibodies have been used to establish and characterize collagen antibody-induced arthritis (CAIA) [19–21]. This model is not dependent on the adaptive immune system but an intact innate immune defense, including activating functional Fc-receptors and complement [22]. Besides, some other factors which affect the susceptibility of the CAIA model are the strain, age, sex of mice and subtype, specificity, and concentration of antibodies [19]. Importantly, so far known antibodies that have the capacity to induce arthritis in mice are all binding to cartilage. The classical antibodies are those binding to conformational (i.e., triple helical) epitopes on CII. More recent studies have shown that antibodies binding to citrullinated peptides that are cross-reacting with CII or citrullinated CII are potently arthritogenic [23, 24]. Antibodies to other cartilage proteins have also been investigated, and it has been found that antibodies to cartilage oligomeric matrix protein can induce arthritis [25]. Arthritis can also be induced with serum from T cell receptor transgenic K/BxN mice, having high titers of antibodies to glucose-6 phosphatase isomerase (G6PI) [26], which specifically target cartilaginous joints because of the deposition of G6PI on the cartilage surface [27]. In addition, we have recently found that antibodies cross-reacting between CII and type XI collagen (CXI) are also highly arthritogenic [28]. These models with arthritis induced by antibodies are all characterized by an acute and self-limited disease, most likely dependent on the half-life of the injected antibodies. Thus, although they are well-characterized and easily controlled models for RA, they do not mimic the more chronic nature of the disease.

To improve the antibody transferred model, we here used cocktails of previously known arthritogenic antibodies [19, 20, 23, 24]. And we selected antibodies to different cartilage proteins for testing the new cocktails to induce more severe and more chronic variants of arthritis. We have also benefited from previous work to identify stimulators/enhancers that could boost arthritis when injected 2–7 days after the injection of antibodies [29, 30]. Screening of TLR and CLR ligands has identified lipopolysaccharide and mannan as the most efficient enhancers of antibody-induced arthritis, but they work in different settings. In mice with mutations of the *Ncf1* gene, leading to a low capacity to induce ROS, mannan injection leads to an antibody-mediated chronic relapsing arthritis [30]. The result is opposite when LPS is given instead of mannan; then, the arthritis developed became milder in a ROS-deficient context [29]. However, *Ncf1* deficiency leads to slightly higher arthritis susceptibility in the antibody phase of arthritis. We have now found that cocktails of antibodies binding to CII, CXI, COMP, and citrullinated CII, and enhanced with mannan or LPS, induced a severe chronic arthritis with bone erosions and activated a new endogenous autoimmune response.

## Methods

### Animal procedures

Founders of DBA/1 and BALB/c mice are from the Jackson Laboratory (Bar Harbor, ME, USA). The B10.Q/rhd substrain (short-named B10Q) is a H2^q^ congenic strain on a C57BL/10 background. Founders of C57BL/6 N (B6/N) mice were from the Jackson Laboratory (Bar Harbor, ME). B10Q.*Ncf1*^m1j^ (short-named BQ.Ncf1*) mice differ by one mutation from B10Q mice, with a non-functional *Ncf1* leading to a deficient reactive oxygen species response. B6/N.Q (short-named B6Q) are mice with a congenic fragment containing the A^q^ gene from the B10.Q mice. The B10Q.*Cia9i* (short-named BQ.*Cia9i*) mice were generated by introgressing *NOD* gene fragment (170.9–173.4 Mb) from non-obese diabetic mouse, containing the *FcγR* gene cluster with FcγR2b, FcγR4, and FcγR3, on the B10.Q genetic background (to be published). In experiments with BQ.*Cia9i* mice, 9–12-week-old male mice were used, whereas with BQ.Ncf1* mice, 12–16-week-old males were used. BALB/c, C57BL/6 J, B6/N.Q, and DBA/1 mice were 9–12-week-old males. All the mice were age-matched, randomized to an experimental group, and investigators were blind to the groups until the end of experiments. All the mice were kept and bred in one unit at the Song Shan Lake Experimental Animal Science and Technology Park, Southern Medical University, Dongguan, China, which is a specific pathogen-free facility having a climate-controlled environment with a 14-h light/10-h dark cycle. The animals were housed in individually ventilated polystyrene cages containing enrichments with standard chow and water given ad libitum. The protocols were approved by local animal welfare authorities.

### Antibody production and purification

The hybridomas were generated and characterized as described elsewhere [27] (Table 1). The antibody-producing hybridomas were selected after subcloning and cultured in ultra-low bovine IgG containing DMEM Glutamax-I culture medium (Gibco BRL, Invitrogen AB, Sweden) with 100 μg/ml of penicillin streptomycin (Sigma, USA). Monoclonal antibodies from the clones were generated from culture supernatant using Integra cell line 1000 flasks (Integra Biosciences, Switzerland) and purified using the Gamma-Bind Plus affinity gel (Pharmacia, Sweden). Antibodies were eluted using 0.1 M glycine-HCl buffer (pH 2.7), neutralized with 1 M Tris–HCl (pH 9.0), and dialyzed extensively against PBS (pH 7.0). The antibody solution was concentrated using a centrifuge device (GE Healthcare, Uppsala, Sweden) with a MWCO of 30 kDa to the desired concentration (15–20 mg/ml) as determined by A_280_ using an extinction coefficient of 1.4. The antibodies were stored freeze-dried. Reconstitution for injection and for keeping the correct osmolarity, sterile water was added to the original volume. The protein concentration was again determined by measuring A_280_, and the solution was sterile filtered using a 0.22-μm dynagard syringe filter (Dynagard, Spectrum Laboratories, CA, USA), aliquoted, and stored at 4 °C or − 20 °C. The limulus amebocyte lysate assay kit and the Pierce™ High Capacity Endotoxin removal Spin Columns (Thermo Fischer Scientific, Waltham, USA) were used following the manufacturer’s instructions to keep the samples to an endotoxin level not higher than 0.1 EU/mg. In order to prepare an antibody cocktail, equal amounts of each antibody were mixed for each experiment.

**Table 1:** Information about the monoclonal antibodies used in this study

Antibody	Epitope	Structure and amino acid sequence	Isotypes	Reference
CIIC1	CII (C1)	358–369: GARGLTGRPGDA	IgG2a	[17]
CIIC2	CII (D3)	687–698: RGAQGPPGATGF	IgG2b	[17]
UL1	CII (U1)	494–504: LVGPRGERGFP	IgG2b	[31]
M2139	CII (J1)	551–564: MPGERRGAAGIAGPK	IgG2b	[32]
L10D9	CXI (D3)		IgG2a	[28]
15A11	COMP	EGF-repeat 4: PSPCHEKADCILERDGSRS	IgG1	[25]
ACC1	CII (CitC1)	CII121-144, CII241-264, CII571-591, CII 916-939 (F4 epitope), CII931-954, C1 (C1-T-CIT365), F4 (F4-T-CIT933), CII571-591	IgG2a	[23]
L243		Anti-HLA DR antibody	IgG2a	ATCC® HB-55™
G11		Anti-human parathyroid cells antibody	IgG2b	[33]
Hy2.15		Anti-hapten antibody	IgG1	[34]

All epitopes are in native triple helical form; amino acids are abbreviated as follows: *G* glycine, *P* proline or hydroxyproline, *E* glutamic acid, *R* arginine, *K* lysine, *H* histidine, *F* phenylalanine, *A* alanine, *L* leucine, *T* threonine, *Y* tyrosine

### Passive transfer of antibodies

Cocktails of the monoclonal antibodies including Cab3, anti-CII antibody cocktail (positive control), and isotype antibody cocktail (negative control) were prepared by mixing equal amounts of each sterile filtered antibody solution to achieve a final concentration of 2 or 4 mg. Mice were injected intravenously (i.v.) with the antibody cocktail (Cab3) composed of M2139, L10D9, and 15A11. For the induction of lpsCAIA (Cab-induced and LPS-enhanced arthritis), the mice received (50 μg/mouse) lipopolysaccharide from *Escherichia coli* O55: B5 (Sigma-Aldrich) intraperitoneally (i.p.) on day 5. For the induction of mCAIA (Cab-induced mannan-enhanced arthritis), the mice received (5 mg/mouse) mannan (Sigma-Aldrich) from *Saccharomyces cerevisiae* i.p. on days 5 and 60. Signs of arthritis in the paws were followed up macroscopically with blind scoring of each red and swollen joint following a previously described protocol [35]. Briefly, macroscopic (clinical) arthritis was defined based on the two following criteria, swelling and redness, and was scored as follows: 1 point was for each inflamed toe or knuckle, whereas 1–5 points were given to an inflamed wrist or ankle according to the severity of disease, resulting in a score of 0 to 15 for each paw and 0 to 60 points for each mouse. Importantly, paws which were still swollen but without erythema were not scored or defined as arthritis.

### Histology and immunofluorescence

To investigate antibody binding to cartilage in vivo, limbs were collected from 9–12-week-old BQ*.Cia9i* mice, which had been injected i.v. with 1 mg of Cab3 containing M2139, L10D9, and 15A11 antibodies; 1 mg of anti-CII antibody cocktail containing M2139, UL1, CIIC1, and CIIC2 antibodies; or 1 mg of isotype antibody cocktail of G11, L243, and Hy2.15 antibodies for each mouse. All of the antibody cocktails injected to animals for immunofluorescence were previously labeled with biotin. To investigate complement activation, 2 mg of Cab3, anti-CII antibody cocktail, or isotype antibody cocktail not labeled with biotin was injected separately. After 48 h, the paws were taken, decalcified, snap frozen, dissected to 7 μm thickness, and stored in − 80 °C until used. For immunohistochemical (IHC) staining, the sections were fixed in acetone on ice for 10 min, followed by air drying for 10 min and hydration in 1× PBS-T for 3 times, 5 min for each time in RT. After blocking with 3% H_2_O_2_ for 10 min, the dissected tissue was further blocked by 5% BSA containing 2% rat sera for 45 min, and then, the sections were incubated with biotin-conjugated goat anti-mouse-C3c antibodies (Nordic MUbio, Heerhugowaard, Netherlands) for 1 h for complement staining. All the sections were incubated with ExtrAvidin™-Peroxidase (Sigma-Aldrich) for 30 min and developed with diaminobenzidine (Vector, CA, USA) for 8–10 min. In addition, sections (7 μm) were stained with hematoxylin/eosin to observe joint morphology. The slides were dried for over 24 h before scanning under inverted microscope (Nikon Corporation, Tokyo, Japan). Sections for confocal studies were incubated with streptavidin-conjugated Alexa Fluor 568 (Thermo Fischer Scientific, Waltham, USA) at 1:300 dilution for 60 min in room temperature and mounted using VECTASHIELD® Mounting Medium with DAPI (Vector, CA, USA). The slides were dried for 30 min before scanning under confocal microscope (LSM 880 with Airyscan, Carl Zeiss, Germany).

For histological assessments, the mouse paws were collected at different time points. The skin was dissected out, and all the paws from mice were cut, fixed, decalcified, dehydrated, and paraffin-embedded. Sections (5–7 μm) were stained using the Hematoxylin and Eosin Staining Kit (Beyotime, Shanghai, China) to observe joint morphology and assess general levels of inflammation. The slides were then scanned using the inverted microscope (Nikon). Pathological scoring of sections for inflammation was performed according to the criteria described previously [29]. Briefly, sections with some infiltrating cells and mild synovitis were given 1 point, and 2 points were given to sections having mild cartilage erosion with moderate synovitis. Sections with severe erosion and destruction of both bone and cartilage having many inflammatory cells as well as severe pannus formation were given 3 points.

### Enzyme-linked immunosorbent assay

To demonstrate cartilage destruction, the concentration of COMP, a biomarker of cartilage degradation, in the serum was measured. Fresh blood was collected from experimental mice (lpsCAIA and mCAIA with BQ.Ncf1* mice) on days 0, 9, 21, 65, and 95 after injection of the antibodies. Sera were prepared and stored at − 20 °C until used. COMP was measured in the serum using an ELISA kit (Cusabio, Wuhan, China) according to the manufacturer’s instructions. The measurement range at the steep part of the standard curve was used for accurate quantification (6.25 to 400 ng/ml), with a minimum detectable dose of mouse COMP less than 1.56 ng/ml.

### Morphology

To confirm the presence of bone erosion and destruction, the hind limbs of the experimental mice were collected (from lpsCAIA and mCAIA with BQ.Ncf1* mice) at the termination period on day 95 and fixed in 4% paraformaldehyde for 24 h. The limbs were scanned by micro-CT (Siemens Inveon, USA). The scanning parameters were set as follows: tube voltage, 80 kVp; electricity, 500 mA; scanning range, 3.078 cm × 3.078 cm; scanning time, 48 min; and scanning resolution, 9.56 μm. To reconstruct and analyze the bone parameters, software OSEM 3D was used.

### Bead-based multiplex immunoassay

Autoantibody responses were analyzed using Luminex technology as described previously [28]. Briefly, all the biotinylated peptides were captured on beads coated with NeutrAvidin (Thermo Fischer Scientific, Waltham, USA). Mouse or human serum samples were diluted 1:100 (v/v) and were prepared to a final concentration of 1 μg/ml in assay buffer (3% BSA, 5% milk powder, 0.1% ProClin300, 0.05% Tween 20, 100 μg/ml NeutrAvidin in PBS) and incubated for 1 h at RT on a shaker. Then, the samples were transferred to a 96-well plate (Greiner Bio-One, Kremsmunster, Austria) containing the peptide-coated beads by manual pipetting. After incubation at RT on a shaker for 75 min, all the beads were washed with 0.05% Tween-20 in PBS (PBST) on a plate washer (EL406, Biotek, Winooski, USA) and then resuspended in a solution containing goat anti-rat IgG Fcγ-PE or anti-human IgG Fcγ-PE (Jackson Immuno Research, West Grove, USA). After 40 min of incubation, the beads were washed with PBS-T and the fluorescence was measured using Luminex FlexMap3D (Luminex, Austin, USA) in median fluorescence intensity (MFI) units. The MFI was used to quantify the interactions of the antibody with the given peptides. The peptides specific for the used antibodies as well as the peptides evoking the highest response among the 72 triple helical CII and COMP peptides tested are shown. The sequences of these peptides are shown in Additional file 1: Table S1.

### Statistical analyses

Quantitative data are expressed as mean ± SEM using the GraphPad Prism version 7 software. For comparison of arthritis severity between groups, including max arthritis score, mean arthritis score, histology score, and the analysis of serum antibody response with Luminex, the two-tailed Mann-Whitney test was used. For multiple comparisons, the one-way ANOVA with Dunn’s multiple comparisons test was used for serum COMP detection. Bone parameters were analyzed using the one-way ANOVA with Tukey’s multiple comparisons test, and the quantitative data are expressed as mean ± SD. *p* values less than 0.05 were considered statistically significant.

## Results

### Autoreactivity of selected cartilage-specific antibodies

**Fig. 1:**
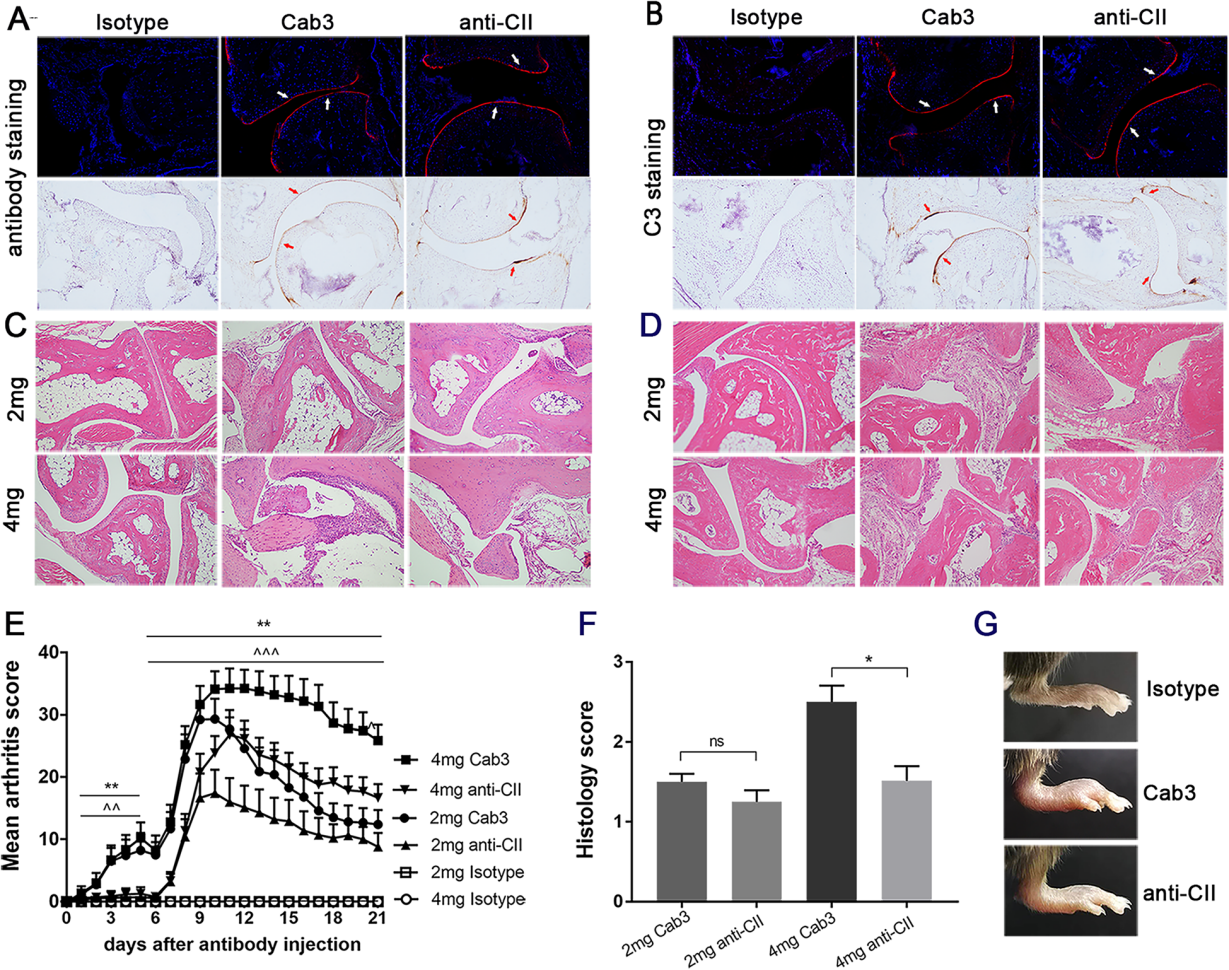
Cartilage-specific antibodies induced arthritis in BQ.*Cia9i* mice. Nine to 12-week-old BQ.*Cia9i* mice were injected with 2 or 4 mg of antibody cocktails. **a** Monoclonal antibody binding to cartilage in vivo. **b** Locally deposited C3 immune complexes are developed on the surface of cartilage within 48 h. **c**, **d** Infiltrating cells, joint surface erosions, and bone destructions on days 5 (**c**) and 21 (**d**) are shown (original mag. × 10). **e** Mean arthritis score (The results are based on combined data from 2 experiments with 5 mice per group. The individual experiment data are shown in Additional file 4: Fig. S1 a and b) and **f** histological scoring of inflammation using a scale of 0–3. **g** Representative pictures of sick paws. 2 mg Cab3 vs 2 mg anti-CII: *p < 0.05; 4 mg Cab3 vs 4 mg anti-CII: ^*p* < 0.05. Values are the mean ± SEM (*n* = 10)

In order to improve the experimental models of antibody-induced arthritis in mice, we selected four different cartilage-binding antibodies: M2139 binding to CII, 15A11 binding to COMP, L10D9 binding to CXI, and ACC1 binding to citrullinated CII (Table 1). These antibodies were selected based on their high efficiency to induce arthritis and that they all specifically bind to cartilage in vivo [23, 25, 28, 36, 37]. The antibodies were prepared and pooled into three different cocktails. Cab3 contains M2139, L10D9, and 15A11 antibodies, and Cab4 consists of M2139, L10D9, 15A11, and ACC1 antibodies, whereas G11, L243, and Hy 2.15 antibodies were used as isotype negative controls. The classical anti-CII antibody cocktail (M2139, UL1, CIIC1, and CIIC2) known to efficiently induce CAIA was used as the positive control [21]. To prove that the antibody cocktails actually bind to cartilage in vivo, we injected biotinylated antibodies into adult mice and isolated the paws 48 h later. The antibody cocktail binding was analyzed by immunohistochemistry and immunofluorescence. Cab3 showed strong staining along the cartilage surface with a similar staining pattern as the classical anti-CII cocktail, whereas isotype control antibodies did not show staining at all. The antibody cocktail binds specifically to cartilage in vivo (Fig. 1a).

### Deposition of complement on the cartilage and development of arthritis

To visualize that the antibody cocktail forms immune complexes containing complement C3 on the cartilage surface, we analyzed joints obtained 2 days after the injection of antibodies. Using antibodies to complement factor C3, we observed strong staining of joints from all of Cab3-injected mice with some infiltrating cells (Fig. 1b). As a comparison, we dissected the joints 5 days after injection of 2 or 4 mg of Cab3, at the time when mice had developed clinical arthritis, and found severe synovitis with inflammatory cell infiltrations and pannus formation (Fig. 1c).

### Development of lpsCAIA with cartilage-specific antibodies

To induce more severe arthritis, we injected BQ.*Cia9i* mice i.v. with Cab3 and 5 days later i.p. with LPS, an enhancer of CAIA [25]. At the time of Cab3 injection within 5 days, the mice had mild arthritis in the front and rear toe joints, and after LPS injection, the severity increased within few days, which peaked around day 10 and lasted until day 21. The Cab3 cocktail caused more severe and chronic arthritis (Fig. 1e) and higher disease incidence (Table 2) than the classical anti-CII cocktail. In addition, Cab3 caused higher numbers of infiltrating cells, and more severe cartilage and bone erosions with 4 mg antibody dose than the anti-CII cocktail on day 21 (Fig. 1d). These observations were confirmed by histological scoring of inflammation (Fig. 1f). The affected joints had classical signs of arthritis such as redness, swelling, and even deformity (Fig. 1g). Mice injected with Cab3 had an earlier and more severe arthritis than mice injected with the anti-CII cocktail even at the early phase of arthritis, before LPS injection.

**Table 2:** Disease incidence and maximum arthritis score in BQ.*Cia9i* mice

Cocktail	B-LPS		A-LPS	
	Incidence	Max arthritis score (mean ± SEM)	Incidence	Max arthritis score (mean ± SEM)
2 mg Cab3	6/10	8.30 ± 2.82*	10/10	31.50 ± 3.22*
2 mg anti-CII	1/10	0.70 ± 0.70*	10/10	20.00 ± 3.16*
4 mg Cab3	8/10	10.40 ± 2.33^^	9/10	36.00 ± 2.80^
4 mg anti-CII	2/10	1.30 ± 1.01^^	10/10	27.90 ± 2.61^

Comparisons were done between 2 mg/4 mg Cab3 and 2 mg/4 mg anti-CII in regard to max arthritis score. Significant difference was observed between two groups. Cab3: M2139 + L10D9 + 15A11; anti-CII: M2139 + UL1 + CIIC1 + CIIC2. *B-LPS* before lipopolysaccharide injection, A-LPS *after lipopolysaccharide injection*. 2 mg Cab3 vs 2 mg anti-CII: *p < 0.05; 4 mg Cab3 vs 4 mg anti-CII: ^*p* < 0.05, ^^*p* < 0.01. The two-tailed Mann-Whitney *U* test was used to calculate the level of significance

### Comparison of lpsCAIA and mCAIA

**Fig. 2:**
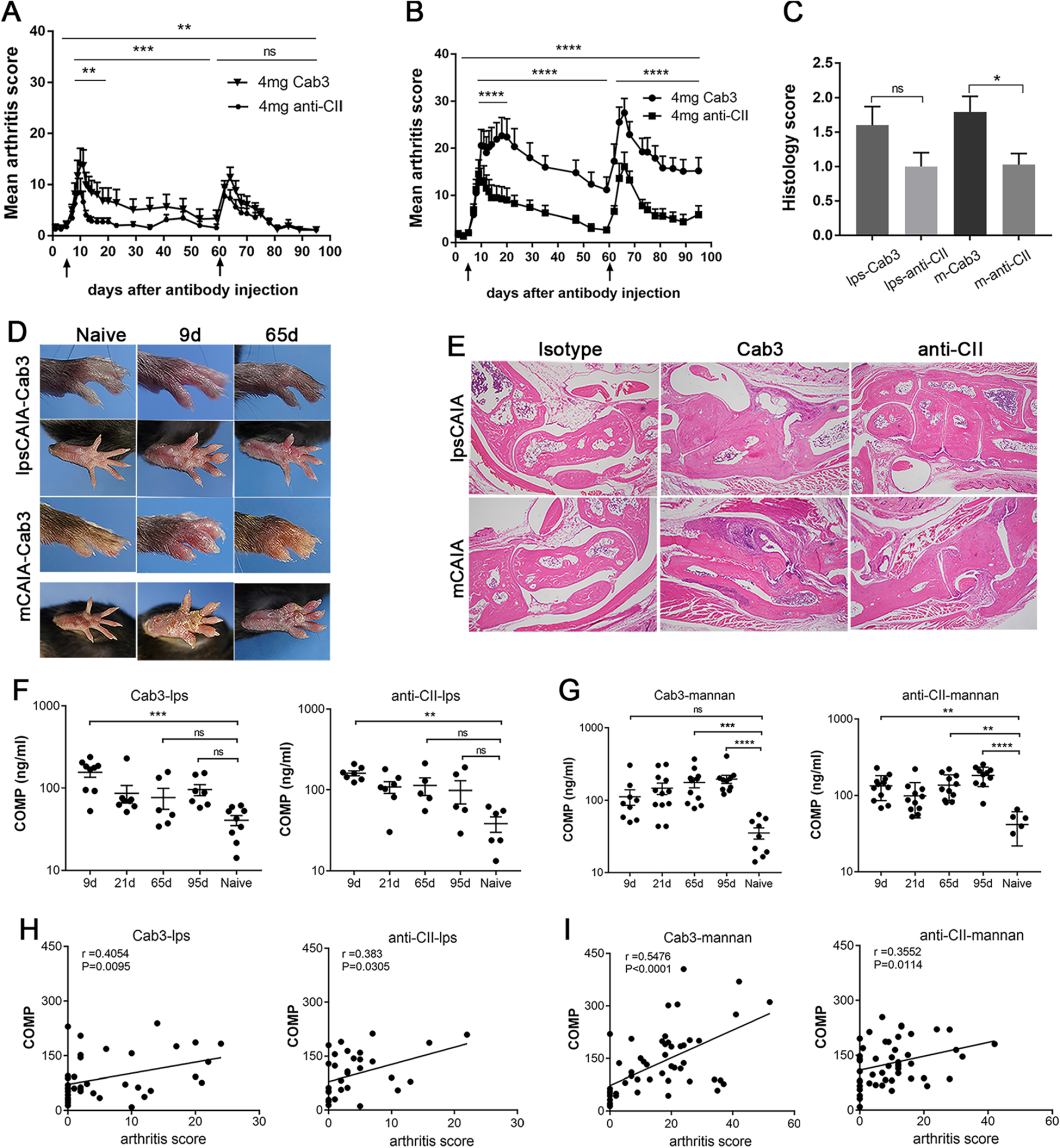
Clinical and histological characteristics of lpsCAIA and mCAIA. Paws from BQ.Ncf1* (12–16-week-old male) mice after injecting 4 mg of Cab3 or anti-CII cocktail were assessed for arthritis development macroscopically. Cell infiltration and bone erosion were evaluated microscopically. **a** Mean arthritis score of lpsCAIA (*n* = 8–10) and **b** mean arthritis score of mCAIA (*n* = 12) after injection of antibody cocktail on day 0 and injection with LPS or mannan on days 5 and 60, as indicated by arrows. **c** H&E-stained paws scored for cell infiltration and bone erosion on a scale of 0–3. **d** Representative images of the paws on days 0, 9, 21, and 65 after injection with Cab3 in lpsCAIA and mCAIA were shown. **e** Histology of joint section (original mag. × 4). **f** Serum COMP concentration of Cab3-induced LPS-enhanced arthritis and anti-CII antibody-induced LPS-enhanced arthritis. **g** Serum COMP concentrations of Cab3-induced mannan-enhanced arthritis and anti-CII-induced mannan-enhanced arthritis. **h**, **i** Correlations between serum COMP to arthritis score in lpsCAIA and mCAIA were analyzed accordingly**.** The data shown as mean ± SEM (*n* = 9–12)

To compare the development of LPS-enhanced and mannan-enhanced arthritis, we injected Cab3 and anti-CII cocktail into BQ.Ncf1* mice. The mice received i.p. injection of either 50 μg of LPS at day 5 (to induce lpsCAIA) or 5 mg of mannan (to induce mCAIA), at days 5 and 60. It is known that the *Ncf1* mutation in the BQ.Ncf1* mice leads to a less severe lpsCAIA but a more severe mCAIA [25, 26]. A more severe and active chronic arthritis was induced in the mCAIA model similar to earlier descriptions [26]. The chronic arthritis was clinically active with relapses and spreading of arthritis to new joints. In both lpsCAIA and mCAIA, the arthritis was more severe if induced with Cab3, compared to anti-CII antibodies (Fig. 2a, b and Additional file 2: Table S2). At the end of the experiments, the hind paws were analyzed histologically, which confirmed the clinical scores (Fig. 2c–e). Isotype control antibodies did not induce arthritis (Additional file 3: Table S3) as expected from previous experiments [16]. To confirm the occurrence of a chronic active destruction of the cartilaginous joints, we measured the levels of circulating COMP, known to be released due to cartilage degradation [38, 39]. The serum COMP in lpsCAIA peaked on day 9 and subsequently decreased gradually (Fig. 2f). In contrast, the serum level of COMP increased persistently even in the late chronic phase of mCAIA (Fig. 2g). The levels of COMP correlated with arthritis severity in both lpsCAIA and mCAIA (Fig. 2h, i).

**Fig. 3:**
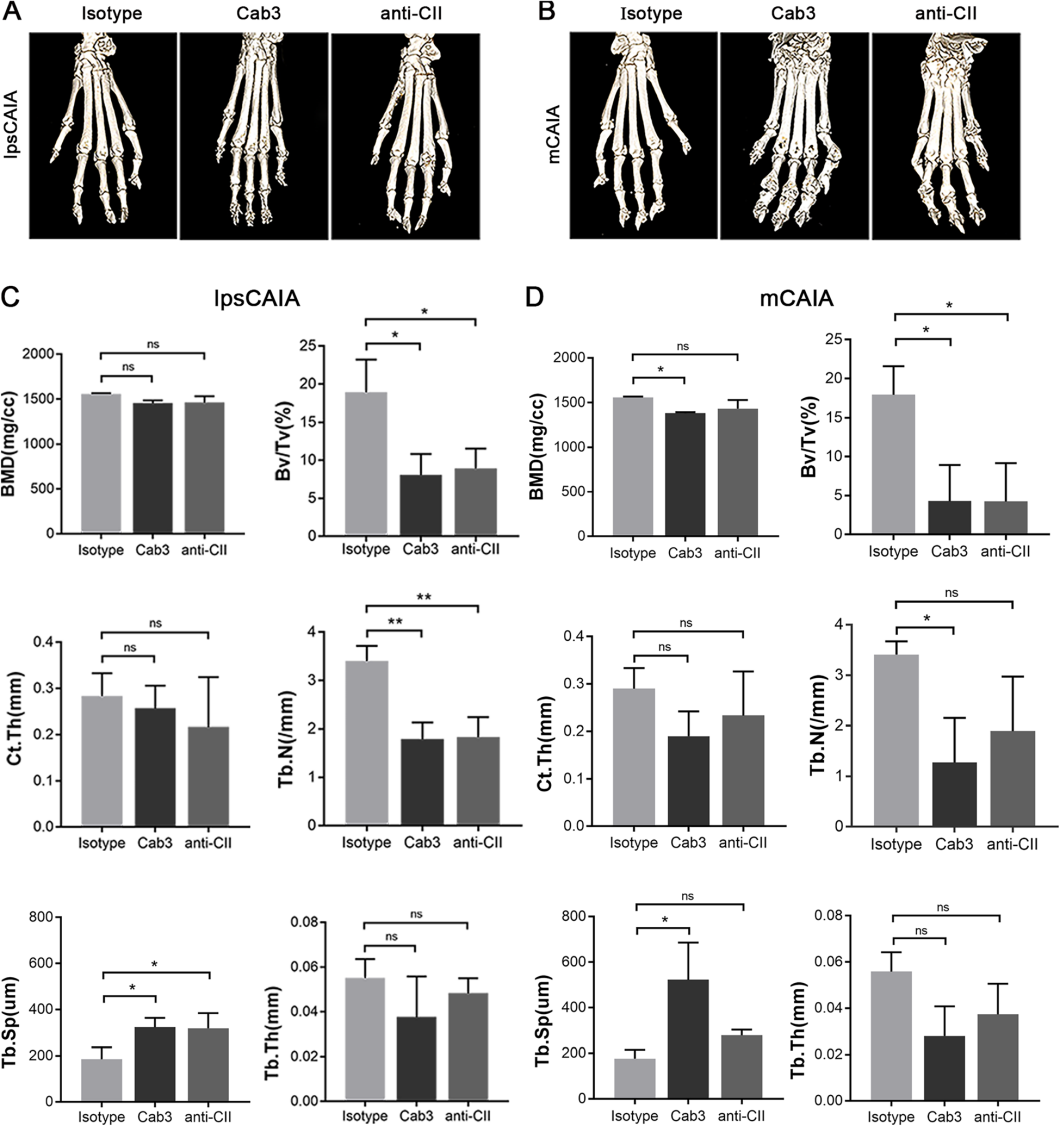
Bone destructions induced by cartilage-binding antibodies. **a**, **b** The representative micro-CT images of isotype control, Cab3, and anti-CII group in lpsCAIA and mCAIA. **c** For the lpsCAIA group, bone mineral density (*p* = 0.07), trabecular bone volume as percent of tissue volume (*p = 0.02), cortical thickness (*p* = 0.90), trabecular number (**p = 0.003), trabecular spacing (*p = 0.04), and trabecular thickness (*p* = 0.26) were analyzed between Cab3 and isotype control (Isotype). **d** For the mCAIA group, bone mineral density (*p = 0.02), trabecular bone volume as a percent of tissue volume (*p = 0.02), cortical thickness (*p* = 0.23), trabecular number (*p = 0.04), trabecular spacing (*p = 0.01), and trabecular thickness (*p* = 0.06) in the distal region of the tibia were analyzed. *p < 0.05; **p < 0.01. The data are shown as mean ± SD (*n* = 3)

The hind paws were subjected to micro-computed tomography (micro-CT) at the end of experiments to analyze the effects of cartilage-binding antibodies on the bone. At this late time point (95 days after the induction), we observed erosions of bone in the joints, concomitantly with joint related new bone formation. In both lpsCAIA and mCAIA groups, the ankle and metacarpophalangeal joints showed more destruction with both Cab3 antibody cocktail and anti-CII antibody cocktail compared to isotype controls (Fig. 3a, b). In addition, we focused on the distal tibia of hind limbs and found some bone parameters including trabecular bone volume and number in Cab3 and anti-CII in lpsCAIA decreased compared to isotype controls, while trabecular spacing was increased. However, bone mineral density, cortical thickness, and trabecular thickness were not affected (Fig. 3c). In the Cab3 mCAIA group, bone mineral density and trabecular bone volume and number were decreased, while trabecular spacing was increased compared to isotype controls but cortical thickness and trabecular thickness were not affected. As for anti-CII cocktail, only trabecular bone volume was decreased compared to isotype controls (Fig. 3d). These results demonstrated a robust effect of cartilage-binding antibodies in lpsCAIA and mCAIA on trabecular bone loss even during the late phase of disease (at day 95).

### Chronic development of arthritis in mCAIA activates an autoimmune response to cartilage

**Fig. 4:**
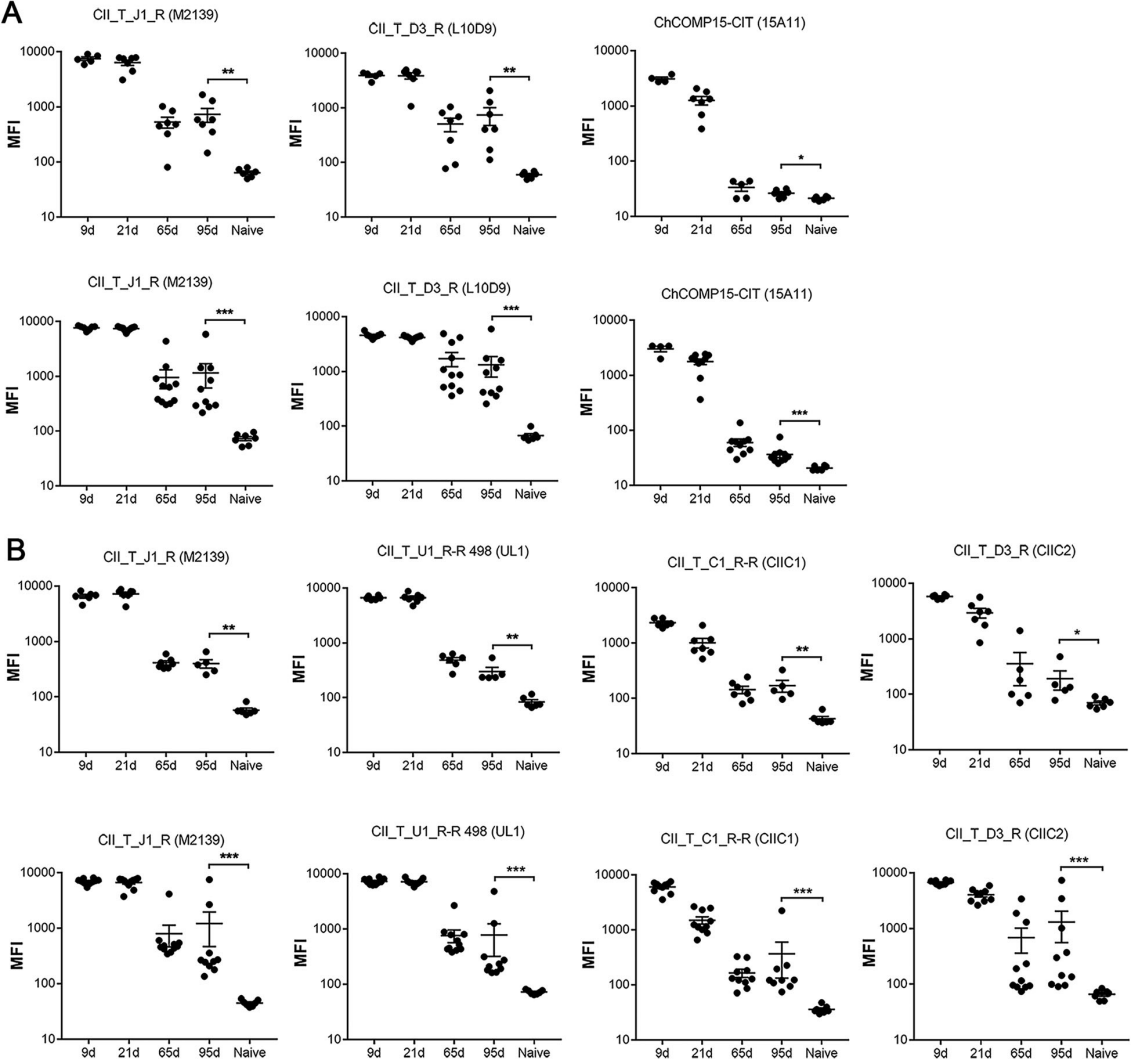
Antibody levels in BQ.Ncf1*mice with lpsCAIA and mCAIA. Measurement of the injected antibodies on days 0, 9, 21, 65, and 95 in lpsCAIA and mCAIA with BQ.Ncf1*mice. **a** Antibodies in Cab3 (M2139, L10D9, and 15A11) cocktail-injected mice bind to J1 (CII_T_J1_R), D3 (CII_T_D3_R), and COMP (ChCOMP15_CIT) epitopes, respectively, in lpsCAIA (above) and mCAIA (below). **b** Antibodies in anti-CII antibody cocktail (M2139, UL1, CIIC1, and CIIC2)-injected mice bind to J1 (CII_T_J1_R), U1 (CII_T_U1 R-R 498), C1 (CII_T_C1_R-R), and D3 (CII_T_D3_R) epitopes, respectively, in lpsCAIA (above) and mCAIA (below). Results show significance on days 0 and 95. The data shown are mean ± SEM

**Fig. 5:**
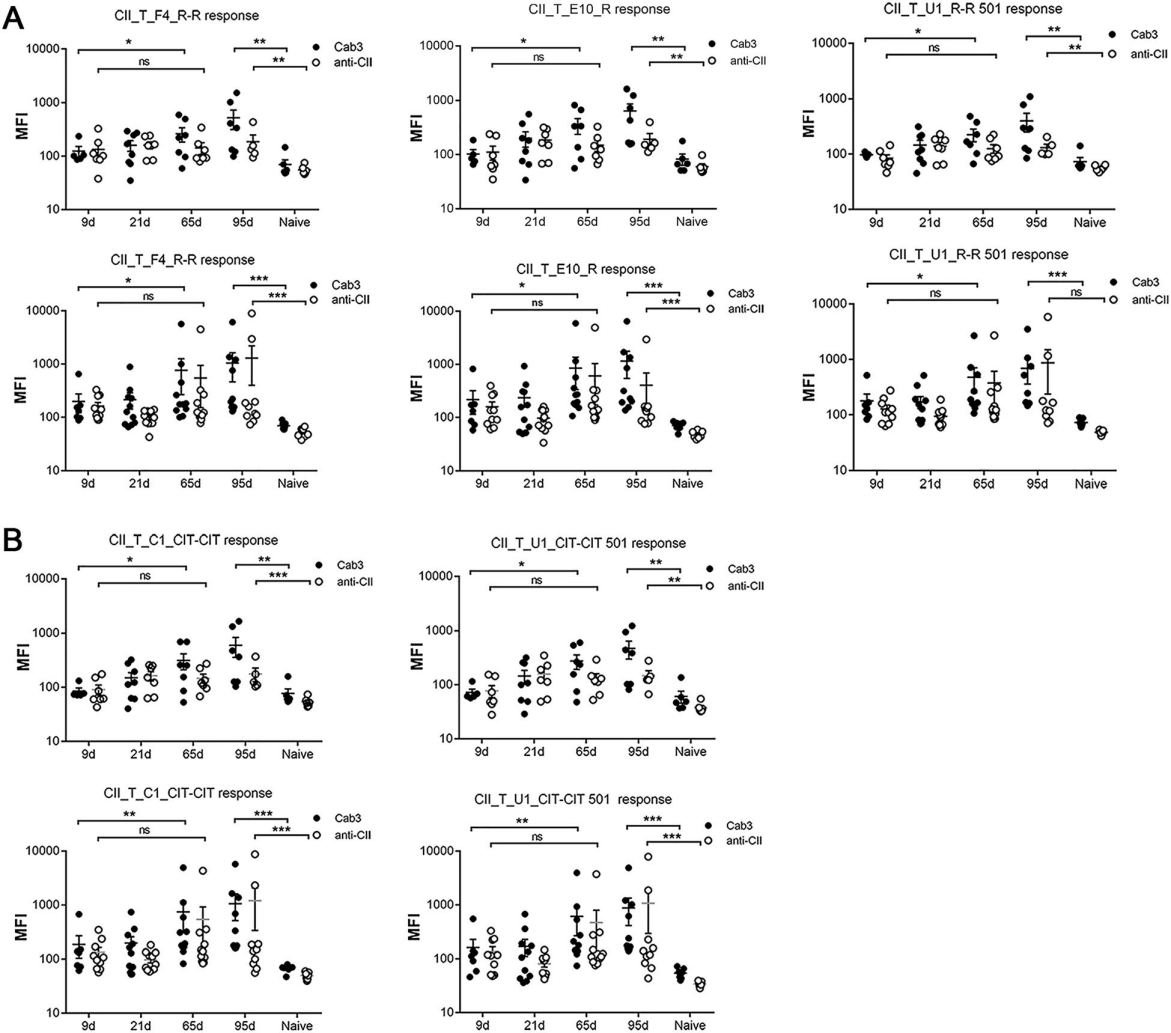
Antibodies to new cartilage epitopes in BQ.Ncf1*mice with lpsCAIA and mCAIA. **a** Antibodies binding specifically to other unmodified epitopes, F4 (CII_T_F4_R-R), E10 (CII_T_E10_R), and U1 (CII_T_U1_R-R 501), in lpsCAIA (above) and mCAIA (below) were detected in Cab3- or anti-CII antibody-injected mice. **b** Antibodies binding specifically to citrullinated epitopes (CII_T_C1_CIT-CIT and C1_T_U1_CIT-CIT) in lpsCAIA (above) and mCAIA (below) were detected in Cab3- or anti-CII antibody-injected mice. The results show significance on days 9 and 95 in the Cab3 group, and on days 0 (naïve) and 95 in both the Cab3 and anti-CII groups. Data shown are mean ± SEM

To address whether the chronic development of arthritis in mCAIA could activate an autoantibody response to cartilage proteins, we used a multiplex Luminex test containing known major triple helical CII, CXI, and COMP epitopes [40]. The injected antibodies were found initially at high levels in the serum, which decreased after few weeks (Fig. 4a, b). However, the antibody levels to all epitopes (J1, D3, and 15A-COMP epitopes in Cab3-injected mice and J1, U1, C1, and D3 epitopes in anti-CII antibody cocktail-injected mice) were still increased above background levels on day 65 after antibody injection. Such an increased level of antibodies to dominant epitopes was still maintained even at day 95 after antibody injection. To investigate whether an immune response to cartilage had been triggered during arthritis development, we analyzed the response to unrelated epitopes on CII and COMP. A small but significantly increased response was noted to both native (Fig. 5a) and citrullinated epitopes (Fig. 5b). Injection of mannan induced a stronger antibody response to cartilage than injection of LPS, possibly due to a stronger chronic arthritis development. On native triple helical CII, the highest response was seen to the F4, E10, C1, and U1 epitopes, all of which are exposed on the cartilage [17, 40, 41]. Interestingly, we also noted an increased response to citrullinated triple helical epitopes. CII on cartilage and components derived from cartilage can be citrullinated in vivo during arthritis, and antibodies to such epitopes can bind to joints in vivo [41, 42]. However, to clearly show that the measured antibodies are specific for citrulline, we need to analyze monoclonal antibodies further [42].

### Efficient induction of arthritis in several mouse strains, including C57BL/6 J mice

**Fig. 6:**
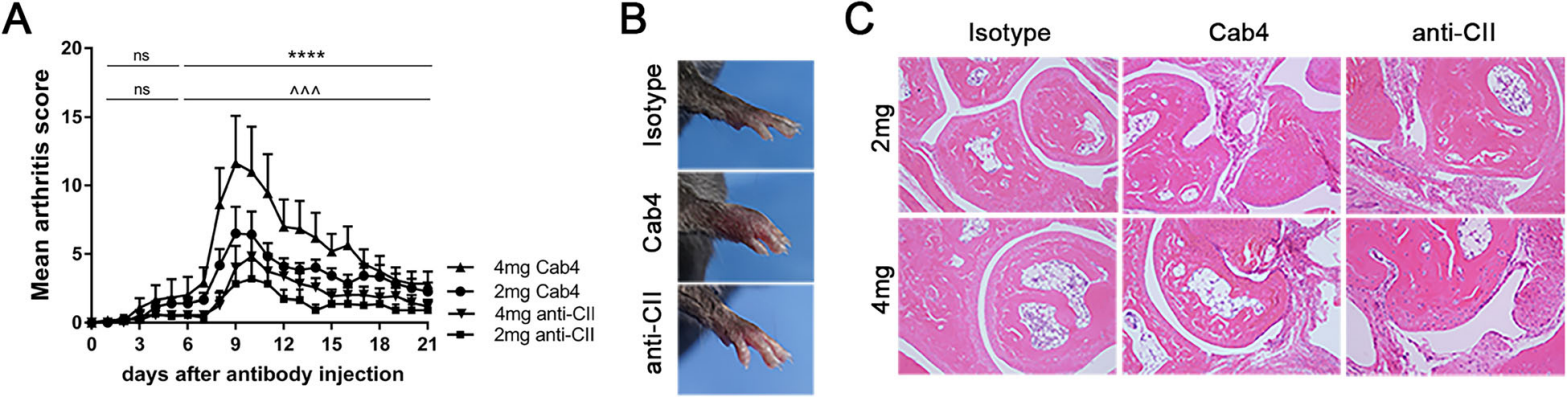
Cab4 monoclonal antibody cocktail induced arthritis in C57BL/6 J mice. **a** Mean arthritis scores (The results are based on combined data from 2 experiments with 5–6 mice per group. The individual experiment data are shown in Additional file 4: Fig. S1 c and d). **b** Representative images of sick paws caused by 4 mg of Cab4 on day 9 are shown. **c** Histological staining of paw sections shows cell infiltrations and bone erosion (original magnification × 10). Values are the mean ± SEM (*n* = 11–12)

To validate the genetic restrictions of the Cab3 cocktail, we tested different mouse strains including BALB/c, DBA/1, C57BL/6 J, and B6/N.Q. The results demonstrated that Cab3 could induce arthritis in all the tested mouse strains, even in the least sensitive C57BL/6J mice, although with only mild arthritis (Tables 3 and 4). To improve the efficiency of the antibody cocktails, we modified the cocktail by including an anti-citrullinated CII monoclonal antibody (ACC1), and this new antibody cocktail is named Cab4. We found that the induction of arthritis with Cab4, following the lpsCAIA protocol, was efficient and had higher incidence and severity with an earlier onset than the classical anti-CII cocktail both before and after the injection of LPS (Fig. 6a–c, Tables 3 and 4).

**Table 3:** Disease incidence and maximum arthritis score in C57BL/6 J mice

Cocktail	B-LPS		A-LPS	
	Incidence	Max arthritis score (mean ± SEM)	Incidence	Max arthritis score (mean ± SEM)
2 mg Cab4	7/12	1.73 ± 0.38	12/12	9.09 ± 1.68*
2 mg Cab3	0/7	0	6/7	6.00 ± 1.86
2 mg anti-CII	4/11	0.91 ± 0.32	6/11	4.27 ± 1.40*
4 mg Cab4	4/11	1.91 ± 1.35	10/11	12.55 ± 3.36
4 mg Cab3	0/7	0	6/7	8.29 ± 3.32
4 mg anti-CII	3/11	0.73 ± 0.41	8/11	5.82 ± 1.55

Comparisons were done between 2 mg/4 mg Cab4 and 2 mg/4 mg anti-CII or between 2 mg/4 mg Cab3 and 2 mg/4 mg anti-CII in regard to max arthritis score. Significant difference was observed between 2 mg Cab4 and 2 mg anti-CII after LPS injection. Cab4: M2139 ± L10D9 ± 15A11 ± ACC1; Cab3: M2139 ± L10D9 ± 15A11; anti-CII: M2139 ± UL1 ± CIIC1 ± CIIC2. 2 mg Cab4 vs 2 mg anti-CII: *p < 0.05. The one-way ANOVA with Dunn’s multiple comparisons test was used to calculate the level of significance

**Table 4:** Disease incidence and maximum arthritis score with common mouse strains

Cocktail	Mice	B-LPS		A-LPS	
		Incidence	Max arthritis score (mean ± SEM)	Incidence	Max arthritis score (mean ± SEM)
2 mg Cab3	DBA/1	4/7	2.00 ± 0.95	7/7	22.86 ± 3.48
	C57BL/6NQ	5/5	6.20 ± 1.50	5/5	28.40 ± 4.23*
	BALB/c	1/7	1.43 ± 1.43	6/7	12.71 ± 4.98*
4 mg Cab3	BALB/c	2/7	1.29 ± 1.13	6/7	20.29 ± 6.26

Comparisons were done in DBA/1, C57BL/6NQ, and Balb/c mice strains injected with 2 mg Cab3 in regard to max arthritis score. Significant difference was observed between C57BL/6NQ and Balb/c mice with 2 mg Cab3 after LPS injection. 2 mg Cab4 vs 2 mg anti-CII: *p < 0.05. The one-way ANOVA with Dunn’s multiple comparisons test was used to calculate the level of significance

## Discussion

We have established new antibody cocktails based on cartilage-specific antibodies and used them to induce severe acute and chronic arthritis, which showed bone erosions and endogenous autoimmune activation to cartilage proteins. The monoclonal antibodies to cartilage proteins, like CII and CXI, COMP, and citrullinated CII, used in this study, are all binding to cartilage in vivo. The three-antibody cocktail (Cab3) could induce arthritis in most of the tested mouse strains, whereas four-antibody cocktail (Cab4) with the addition of ACC1 was more efficient than Cab3 in the C57BL/6 strain. Both Cab3 and Cab4 induced mild arthritis few days after the injection, but the development of arthritis could be enhanced by giving an i.p. injection of LPS or mannan, using earlier reported protocols [26, 30]. Thus, three different diseases could be induced with the Cab antibodies: (1) cartilage antibody-induced arthritis (CAIA), (2) LPS-enhanced arthritis (lpsCAIA), and (3) mannan-enhanced arthritis (mCAIA). These diseases differ in genetic restrictions and pathogenesis. This is best demonstrated by the use of a mouse strain with a mutation in the *Ncf1* gene, leading to a lower reactive oxygen species (ROS) response. The CAIA is slightly enhanced in disease severity and incidence in the presence of the *Ncf1* mutation whereas the opposite is seen after an injection of LPS (lpsCAIA), demonstrating that they are caused by different mechanisms. The enhancing effect on arthritis severity after the induction of arthritis by LPS could be explained by the need of the neutrophils to use ROS to mediate toxic, inflammatory effects. In the mCAIA model, the presence of the *Ncf1* mutation has a more dramatic effect and enhanced the development of arthritis, allowing it to develop into a chronic relapsing disease [26]. Importantly, we observed that the chronic phase in both LPS-enhanced and mannan-enhanced arthritis involves significant bone erosion, similar to RA. The same specificity of antibodies can also be observed in patients with RA, and these epitopes are highly conserved between mouse and humans [43].

Only antibodies binding to the cartilage or the cartilage surface (CII, citrullinated CII, CII/CXI, COMP, and G6PI) have so far been shown to induce arthritis in mice [15, 18–21, 23, 39]. The pathogenic mechanisms are partly known [1, 22]. The antibodies bind to cartilage within minutes after injection, subsequently destabilizing the cartilage and forming local immune complexes. The immune complexes bind to the Fc receptor expressing cells, and the earliest symptom of the mouse is pain induction leading to a different behavior [41, 44]. Subsequently, local macrophages will be activated but also infiltration of inflammatory cells consisting of a few T cells and macrophages. If injected with LPS, a dramatic influx of neutrophils is seen with the development of severe arthritis. If injected with mannan, chronic arthritis driven by macrophages develops [30]. The Cab cocktail seemed more efficient than previous protocols, but an important question is if there are differences between antibodies of different epitope specificities [45]. In susceptible strains and with a higher dose (4 mg), the antibodies could induce arthritis by themselves and it will be of interest to analyze the unique type of pathogenic effect they induce in a comparative analysis. As earlier reported, the chronic development of arthritis developed even though most of the injected pathogenic antibodies disappeared [30]. Interestingly, we discovered that bone erosions and induction of autoantibodies to new cartilage epitopes developed during the chronic phase of both LPS-induced and mannan-induced models. The autoimmune activation is likely due to further exposure of cartilage proteins during inflammation. Whether this in turn contributes to the chronic development of arthritis is however not clear, but interestingly, the specificity of the antibody responses evoked is known to have both pathogenic and protective functions [42].

These models (CAIA, lpsCAIA, and mCAIA) seem to mimic certain aspects of RA pathogenesis and are more defined as alternative to commonly used models, which are often more variable and possibly representing other aspects of RA. Examples of such models are collagen-induced arthritis or spontaneous arthritis in the SKG, K/BxN, or *Ncf1* mutation mouse strains [5, 21, 45]. Induction of the new Cab3-induced model is rapid and results in a synchronized, steady, and controlled disease progression that exhibits histological similarities to the classic CIA model. Most importantly, models are established, which involves targeting of more joint molecules making the inflammatory response more destructive and chronic and initiating a self-perpetuating process with the involvement of an immune response to endogenous cartilage. Therefore, it allows studies of different pathways depending on which enhancers are used after the injection of the antibodies as well as which mouse strains are used. Besides, these models are useful for studies to analyze the pathogenesis of arthritis and the function of antibodies and adjuvants in vivo as well as for screening and validating pharmacological agents.

## Conclusions

It is shown that the new arthritis model induced with a cocktail of cartilage binding antibodies involves not only severe acute arthritis but also a chronic active disease course, which with bone erosions and endogenous autoimmune activation to cartilage proteins. The antibodies induced arthritis after a few days, and the disease could be severely enhanced and give different types of chronic disease depending on whether the direction of the disease is promoted by LPS or mannan. The new model will be useful as it mimics RA regarding chronicity and bone erosions, it allows induction of different types of arthritis by injecting different adjuvants such as LPS or mannan, and it is possible to use in relatively arthritis-resistant strains such as the C57BL/6 J mice.

## Supplementary information

**Additional file 1 : Table S1.** Showing information about peptides sequences. The cyclic peptide \P6-R247 (^239^CHADSVLERDGSRSSVC^255^) shares the same core sequence with P6-R-R but contains additional Cys residues at both N and C terminus to facilitate the formation of an intra-chain disulfide bond and thus a cyclic form of the peptide in neutral buffer solution. c=citrulline.

**Additional file 2 **: **Table S2**. Showing disease incidence and maximum arthritis score in BQ.Ncf1* mice. Comparisons were done between 2mg/4mg Cab3 and 2mg/4mg anti-CII in regard of max arthritis score. Significant difference was observed between 4mg Cab3 and 4mg anti-CII after LPS injection.

**Additional file 3 : Table S3.** Showing disease incidence and maximum arthritis score with isotype control antibodies. Isotype controls: G11 + L243 + Hy2.15; Mice: 12-weeks-old males; 5-6 mice/group. B-LPS: before lipopolysaccharide injection; A-LPS: after lipopolysaccharide injection.

**Additional file 4 : Figure S1.** Showing the individual experimental data of monoclonal antibody cocktails inducing arthritis in BQ.*Cia9i* mice (a, b) and C57BL/6J mice (c, d). 2 mg Cab3 or Cab4 vs 2 mg anti-CII: *, *p* < 0.05; 4 mg Cab3 or Cab4 vs 4 mg anti-CII: ^, *p* < 0.05. Values are the mean + SEM (*n* = 5~6).

## Data Availability

All data supporting our findings are shown in the article or in the additional files.

## Abbreviations

ACPAs: Anti-citrullinated protein antibodies
BMD: Bone mineral density
Bv/Tv: Trabecular bone volume as percent of tissue volume
CII: Collagen type II
CXI: Collagen type XI
COMP: Cartilage oligomeric matrix protein
CIA: Collagen-induced arthritis
Cab3: Three cartilage-binding antibodies
CAIA: Collagen or cartilage-binding antibody-induced arthritis
lpsCAIA: Cab3-induced LPS-enhanced arthritis
mCAIA: Cab3-induced mannan-enhanced arthritis
Ct. Th: Cortical wall thickness
EU: Endotoxin unit
FcγR: Fc gamma receptor
G6PI: Glucose-6-phosphate isomerase
GIA: Glucose-6-phosphate isomerase peptide induced arthritis
i.v.: Intravenously
i.p.: Intraperitoneally
IHC: Immunohistochemical
LPS: Lipopolysaccharide
mAbs: Monoclonal antibodies
MIP: Mannan-induced psoriasis
micro-CT: Micro-computed tomography
Ncf1: Neutrophil cytosolic factor 1
NOX2: NADPH oxidase 2
PsA: Psoriatic arthritis
PBS: Phosphate-buffered saline
RF: Rheumatoid factors
RA: Rheumatoid arthritis
SLE: Systemic lupus erythematosus
ROS: Reactive oxygen species
SNP: Single-nucleotide polymorphism
TLR: Toll-like receptor
CLR: C-type lectin receptor
Tb. Th: Trabecular thickness
Tb. N: Trabecular number
Tb. Sp: Trabecular separation
