# Supplementary material for: Cartilage-binding antibodies initiate joint inflammation and promote chronic erosive arthritis
Source: Arthritis Res Ther. 2020 May 24;22:120. doi: 10.1186/s13075-020-02169-0 (PMC7245816; doi:10.1186/s13075-020-02169-0)
Supplement: Supplementary file 1 — Additional file 1 : Table S1. Showing information about peptides sequences. The cyclic peptide \P6-R247 (239CHADSVLERDGSRSSVC255) shares the same core sequence with P6-R-R but contains additional Cys residues at both N and C terminus to facilitate the formation of an intra-chain disulfide bond and thus a cyclic form of the peptide in neutral buffer solution. c=citrulline. [file 13075_2020_2169_MOESM1_ESM.docx]

**Additional file 1: Table S1** Information about peptides sequence

| Peptides | Epitopes | | Sequences | |  |
| --- | --- | --- | --- | --- | --- |
| CII_T_J1_R | | J1 (541-564) | | (GPp)5-GAQGPpGLQGMpGERGAAGIAGPK-(GPp)5-branch-biotin | |
| CII_T_D3_R | | D3 (676-699) | | (GPp)5-GPTGVTGPKGARGAQGPpGATGFp-(GPp)5-branch-biotin | |
| ChCOMP 15-CIT | | P6 (240-254) | | (GPp)5-CHADSVLEcDGScSSVC-(GPp)5-branch-biotin | |
| CII_T_U1_R-R 498 | | U1 (493-504)) | | (GPp)5-GLVGPRGERGFp-(GPp)5-branch-biotin | |
| CII_T_C1_R-R | | C1 (345-369) | | (GPp)5-GDpGRpGEpGLpGARGLTGRpGDA-(GPp)5-branch-biotin | |
| CII_T_F4_R-R | | F4 (916-939) | | (GPp)5-GDKGEAGEpGERGLKGHRGFTGLQ-(GPp)5-branch-biotin | |
| CII_T_E10_R | | E10 (766-789) | | (GPp)5-GAEGPpGPQGLAGQRGIVGLpGQR-(GPp)5-branch-biotin | |
| CII_T_U1_R-R 501 | | U1 (496-507) | | (GPp)5-GPRGERGFpGER-(GPp)5-branch-biotin | |
| CII_T_C1_CIT-CIT | | C1 (345-369) | | (GPp)5-GDpGcpGEpGLpGAcGLTGcpGDA-(GPp)5-branch-biotin | |
| CII_T_U1_CIT-CIT | | U1 (481-504) | | (GPp)5-GDQGVpGEAGApGLVGPcGEcGFp-(GPp)5-branch-biotin | |

The cyclic peptide P6-R247 (^239^CHADSVLERDGSRSSVC^255^) shares the same core sequence with P6-R-R but contains additional Cys residues at both N and C terminus to facilitate the formation of an intra-chain di-sulfide bond and thus a cyclic form of the peptide in neutral buffer solution. c= citrulline.
