# Supplementary material for: Cartilage-binding antibodies initiate joint inflammation and promote chronic erosive arthritis
Source: Arthritis Res Ther. 2020 May 24;22:120. doi: 10.1186/s13075-020-02169-0 (PMC7245816; doi:10.1186/s13075-020-02169-0)
Supplement: Supplementary file 2 — Additional file 2 : Table S2. Showing disease incidence and maximum arthritis score in BQ.Ncf1* mice. Comparisons were done between 2mg/4mg Cab3 and 2mg/4mg anti-CII in regard of max arthritis score. Significant difference was observed between 4mg Cab3 and 4mg anti-CII after LPS injection. [file 13075_2020_2169_MOESM2_ESM.docx]

**Additional file 2: Table S2** Disease incidence and maximum arthritis score in BQ.*Ncf1** mice

| **Cocktail** | **Incidence** | | **Max arthritis score (mean + SEM)** | **Incidence** | **Max arthritis score (mean + SEM)** |
| --- | --- | --- | --- | --- | --- |
|  | **B-LPS** | | | **A-LPS** | |
| 2mg Cab3 | 7/7 | 7.14 + 2.37 | | 7/7 | 17.29 + 3.30 |
| 2mg anti-CII | 4/7 | 4.57 + 1.88 | | 6/7 | 12.57 + 2.60 |
| 4mg Cab3 | 10/11 | 7.18 + 1.45 | | 11/11 | 14.73 + 2.50^ |
| 4mg anti-CII | 10/11 | 4.73 + 1.77 | | 10/11 | 7.18 + 2.17^ |
|  | **B-Mannan** | | | **A-Mannan** | |
| 2 mg Cab3 | 6/7 | 4.00 + 1.27 | | 7/7 | 12.86 + 1.24 |
| 2 mg anti-CII | 7/7 | 5.57 + 1.09 | | 7/7 | 14.00 + 1.60 |
| 4 mg Cab3 | 11/12 | 2.67 + 0.72 | | 12/12 | 29.33 + 3.37 |
| 4 mg anti-CII | 9/12 | 3.25 + 0.69 | | 11/12 | 20.67 + 3.19 |

Comparisons were done between 2mg/4mg Cab3 and 2mg/4mg anti-CII in regard of max arthritis score. Significant difference was observed between 4mg Cab3 and 4mg anti-CII after LPS injection. B-Mannan: before mannan injection; A-Mannan: after mannan injection. The Two-tailed Mann–Whitney test was used to calculate the level of significance.
