# Supplementary material for: Cartilage-binding antibodies initiate joint inflammation and promote chronic erosive arthritis
Source: Arthritis Res Ther. 2020 May 24;22:120. doi: 10.1186/s13075-020-02169-0 (PMC7245816; doi:10.1186/s13075-020-02169-0)
Supplement: Supplementary file 3 — Additional file 3 : Table S3. Showing disease incidence and maximum arthritis score with isotype control antibodies. Isotype controls: G11 + L243 + Hy2.15; Mice: 12-weeks-old males; 5-6 mice/group. B-LPS: before lipopolysaccharide injection; A-LPS: after lipopolysaccharide injection. [file 13075_2020_2169_MOESM3_ESM.docx]

**Additional file 3: Table S3** Disease incidence and maximum arthritis score with isotype control antibodies

| 4 mg Isotype | lpsCAIA | | | | mCAIA | | | |
| --- | --- | --- | --- | --- | --- | --- | --- | --- |
|  | Incidence | | Max arthritis score (mean + SEM) | | Incidence | | Max arthritis score (mean + SEM) | |
|  | B-LPS | A-LPS | B-LPS | A-LPS | B-LPS | A-LPS | B-LPS | A-LPS |
| BALB/c | 0/6 | 0/6 | 0 | 0 | - | - | - | - |
| BQ.*Cia9i* | 0/5 | 0/5 | 0 | 0 | - | - | - | - |
| BQ.*Ncf1** | 0/6 | 0/6 | 0 | 0 | 0 | 3/6 | 0 | 2.17 + 1.11 |

Isotype controls: G11 + L243 + Hy2.15；Mice: 12-weeks-old males; 5 - 6 mice/group.
