# Supplementary figures and images for: Cartilage-binding antibodies initiate joint inflammation and promote chronic erosive arthritis
Source: Arthritis Res Ther. 2020 May 24;22:120. doi: 10.1186/s13075-020-02169-0 (PMC7245816; doi:10.1186/s13075-020-02169-0)

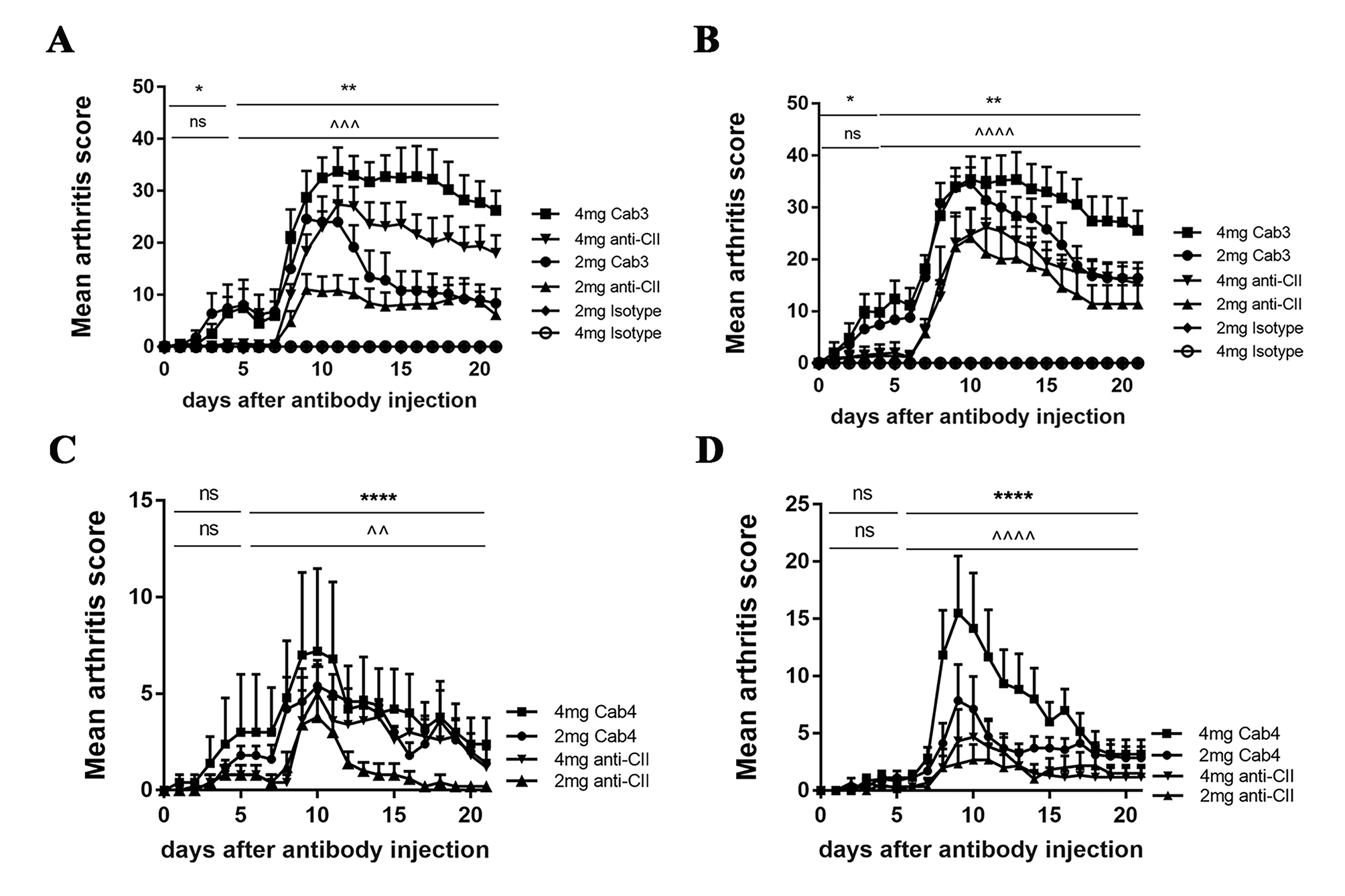

Supplement: Supplementary file 4 — Additional file 4 : Figure S1. Showing the individual experimental data of monoclonal antibody cocktails inducing arthritis in BQ.Cia9i mice (a, b) and C57BL/6J mice (c, d). 2 mg Cab3 or Cab4 vs 2 mg anti-CII: *, p < 0.05; 4 mg Cab3 or Cab4 vs 4 mg anti-CII: ^, p < 0.05. Values are the mean + SEM (n = 5~6). [file 13075_2020_2169_MOESM4_ESM.tif]
